# Attending to Visual Stimuli versus Performing Visual Imagery as a Control Strategy for EEG-based Brain-Computer Interfaces

**DOI:** 10.1038/s41598-018-31472-9

**Published:** 2018-09-05

**Authors:** Nataliya Kosmyna, Jussi T. Lindgren, Anatole Lécuyer

**Affiliations:** 1 MIT Media Lab, 75 Amherst St, Cambridge, MA 02139 USA; 2 Inria Rennes, 263 Avenue General Leclerc, Rennes, 35042 France

## Abstract

Currently the most common imagery task used in Brain-Computer Interfaces (BCIs) is motor imagery, asking a user to imagine moving a part of the body. This study investigates the possibility to build BCIs based on another kind of mental imagery, namely “visual imagery”. We study to what extent can we distinguish alternative mental processes of observing visual stimuli and imagining it to obtain EEG-based BCIs. Per trial, we instructed each of 26 users who participated in the study to observe a visual cue of one of two predefined images (a flower or a hammer) and then imagine the same cue, followed by rest. We investigated if we can differentiate between the different subtrial types from the EEG alone, as well as detect which image was shown in the trial. We obtained the following classifier performances: (i) visual imagery vs. visual observation task (71% of classification accuracy), (ii) visual observation task towards different visual stimuli (classifying one observation cue versus another observation cue with an accuracy of 61%) and (iii) resting vs. observation/imagery (77% of accuracy between imagery task versus resting state, and the accuracy of 75% between observation task versus resting state). Our results show that the presence of visual imagery and specifically related alpha power changes are useful to broaden the range of BCI control strategies.

## Introduction

A brain-computer interface (BCI) typically translates the electrophysiological brain signals into an output that reflects the user’s intent or mental activity. BCIs can be beneficial to people with severe motor disabilities^1^ and may also be used beyond the scope of medical applications^2,3^. BCI is not a perfectly accurate technology as it suffers from numerous issues, namely it is known to be a difficult task to reliably discriminate brain signal patterns from every subject. There are different approaches to improve the performance of BCIs. Most studies focus on signal processing and classification aspects. However, BCI performance can also be improved by optimizing the user’s control strategies and therefore by identifying new and efficient mental tasks to achieve reliable control^4^.

Nowadays, the most common imagery task used in BCI is motor imagery, asking a user to imagine moving a specific part of the body such as a hand or a foot^5^. It is used to control assistive technologies^6^, or even computer games^7^. However, motor imagery based BCIs are seldom used outside laboratories due to their lack of reliability. The following main findings can be highlighted. First, around 20% of the BCI users are not able to obtain effective control. This phenomenon is being sometimes referred to as “BCI illiteracy”^8^. Second, for even the remaining 80% of the people, motor imagery might not be the best mental task to control a BCI (not very intuitive for people with motor disabilities). One option is to explore other mental strategies for BCI control. A broad set of possibilities is proposed in the literature, such as imagining music^9^, phoneme imagery^10^, visual imagery of faces^11^, mental rotation and word association^4^.

Here we are interested in mental tasks that have not been much tested yet for controlling a BCI, namely visual imagery of objects. We focus on visualization in this work because we respond to and process visual data better than any other type of data: the human brain processes images 60,000 times faster than text^12^, and 90 percent of information transmitted to the brain is visual^13^. Since we are visual by nature, and given how important the visual modality is, we propose to use this skill to design control strategies for BCIs.

For our study we chose two visual stimuli: a flower and a hammer. However there is a challenging problem to interpret the performance of visual imagery (and any type of mental imagery in general): some participants may show good performance but which is due to resorting to other strategies that do not involve imagery in the target modality^14^. Assessing mental imagery and the ability to distinguish between the different types of imagery thus requires a carefully designed experimental paradigm. For this end, we performed a study where imagery is guided by previously perceived stimuli. This also provides EEG data during visual perception focused on cued image, data that can be used to study differences in brain activity between perception and imagery processes.

## Visual Imagery

Visual imagery is defined as “the manipulation of visual information that comes not from perception but from memory”^15^. Visual imagery has been mostly studied with PET or fMRI. However, PET and fMRI require sitting/laying down in the scanner, which may not be realistic for some human-computer interaction settings. Another disadvantage for using PET lies in the fact that it involves injection of a radioactive material into the blood. The other problem is that although PET, similarly to fMRI has excellent spatial resolution, the delay between the shift in activity and the resulting change in blood flow is between 6 and 9 seconds (one can interpret where the event occurred but not be sure of exactly when it occurred, which makes statements about the relative order of activations problematic). These acquisition tools are quite expensive, and not portable, which also limits their use cases. In our work we will *focus on the EEG-based BCI systems* as the currently most affordable ones for research labs and centers. We are particularly interested if EEG can provide evidence of visual imagery to achieve desirable end-user BCI applications, as EEG-based BCIs give a possibility of independent use, set-up simplicity and wearability.

## Visual Observation and Visual Imagery using EEG-based BCIs

Visual observation (sometimes “attention” in the literature) has been used with EEG-based BCIs to test if different classes of stimuli, such as cars, mushrooms, chairs, shoes, animals, etc., evoke spatially and temporally different responses when observing the images^16–18^.

In their work, Shenoy and Tan^19^ explore whether the Event Related Potential (ERP) features that encode object category can be used to label images on a single-trial basis, without explicitly requiring users to consciously categorize the images but just to observe them. They perform an experiment where they show three different categories of images: faces, inanimate objects and animals, where the discrimination was achieved at more than 65 percent of accuracy. Simanova *et al*.^20^. build an offline BCI system based on conceptual category detection by studying event-related potential (ERP) activations. They show that it is possible to get a system that allows detecting the semantic categories of a few concrete concepts (e.g. a lamp, a chair).

Another work by Kosmyna *et al*.^21^ builds on Simanova’s *et al*. study and explores further whether it is possible to build and asynchronous ERP-based BCI to distinguish between objects of similar and dissimilar “conceptual” categories. They obtained classification accuracies around 65% for similar conceptual categories and around 70% for conceptually distinct categories. Several studies reported that similar patterns of brain activity are observed when perceiving objects^19,22–25^.

However, all the aforementioned studies focus on asking the participants to observe the objects on the images and there are almost no studies that investigate visual imagery of actual objects on the pictures using EEG. Moreover, we were interested in investigating visual imagery as a control paradigm to be used in offline and/ir online BCIs, thus for this study we chose two images which could be considered as “ecological” and “user friendly” (images coming from the environment and not generated artificially) and they represent actual, real objects: a flower and a hammer. We include visual observation task in our experimental set-up to have a comparison with the state of the art on visual observation. We also include ERP analysis as well as one of the mostly well-studied brain activity that is used to analyse mental imagery - alpha rhythms.

## Manifestation of Mental Imagery in EEG recordings: Alpha Oscillations

The most commonly measured rhythm in the human EEG is the alpha rhythm, generally referring to the frequency band spanning from 8 to 12 Hz. For a long time the alpha activity was reported to be strongest when people keep their eyes closed and rest, and it was known as an <<idling rhythm>>^26^. Over the last 20 years, this <<idling>> hypothesis has been revisited. A lot of studies report that “alpha activity can increase with cognitive load”^27,28^. Ray and Cole^29,30^ found increased alpha power in a mental imagery task, particularly at parietal sites. Klinger *et al*.^31^ observed similar findings of increased alpha during imagery process.

Klimesch and colleagues^32^ introduced a theory about a wider role of alpha oscillatory networks in cognitive processes that involve attention and memory. The main principle is that “activation of this network leads to alpha desynchronization and the greater task demands are within the network, the more inhibition is needed, thus inducing alpha synchronization” meaning that alpha activity is higher with the cognitive load related to the task at hand difficulty increasing. Cooper *et al*.^33^ showed that alpha synchronization is related to internally versus externally directed attention. They presented sequences of stimuli in the visual, auditory and tactile domains and then asked their participants to imagine the sequences. They found that “alpha activity was higher during the imagery of stimulus sequences (i.e., internally directed attention) than during their presentation (i.e., externally directed attention)”. In these studies the observed synchronization of alpha activity has been interpreted to reflect “selective inhibition of task-irrelevant brain areas or inhibition of interfering external input”^34,35^, and to reflect “internal information processing involving top-down control of internally represented information” (e.g.^36,37^). The increase of alpha power over regions processing the distracting information was reported by Frey *et al*.^38^. In this study the participants were required to maintain the “representation of the face identities versus face orientation, and more alpha power was observed over the ventral visual stream”.

## Hypotheses

Based on the mentioned literature, we can conclude that up to now, very few studies proposed investigating the plausibility of using visual imagery as a paradigm for EEG-based BCIs. The objective of the current study is to examine if differences are present between the two visual imagery conditions in general. Moreover we investigate, if it is possible to distinguish between imagery and observation conditions. Finally, we test if we are able to differentiate the imagery condition from the “resting” state, e.g. when users are not controlling their brain activity in order to generate any particular brain pattern. Almost each BCI system nowadays uses this state, mostly to provide the users a break between the trials. However, several papers^39,40^ use the resting state as an actual class for comparing it to the other mental imagery action. They evoke the ease of the designing of a BCI system, when considering resting state: the user needs to produce the desired brain pattern intentionally only during the periods in which he/she wants to activate the BCI, and he/she can remain in resting state at all other times.

We study the following null hypotheses that based on EEG,

H1. visual observation and imagery tasks cannot be differentiated,

H2. imagery of different visual stimuli cannot be differentiated,

H3. observation task towards different visual stimuli cannot be differentiated,

H4. observation or imagery tasks cannot be not differentiated from the rest state.

## Results

We first present the obtained results on the observation tasks of two stimuli and then we discuss the results on imagery tasks. We refer to the observation task and the imagery task as “observation” and “imagery” when we talk about 2-class classification problem. We refer to observation task and imagery task as “observation-merged” and “imagery-merged” when we take a union of two tasks, e.g., we consider a classification result for observation task for the two stimuli that were present as one task and one result. This means that we collected all the trials regardless of stimuli (hammer or flower) where the task is to imagine as trials representing the ‘imagery-merged’ class. We do likewise for the ‘observation-merged’ class. Hence, the classification problem will still remain a 2 class problem, but there will be more trials per class.

### Classification of observation stimuli

**Figure 1:**
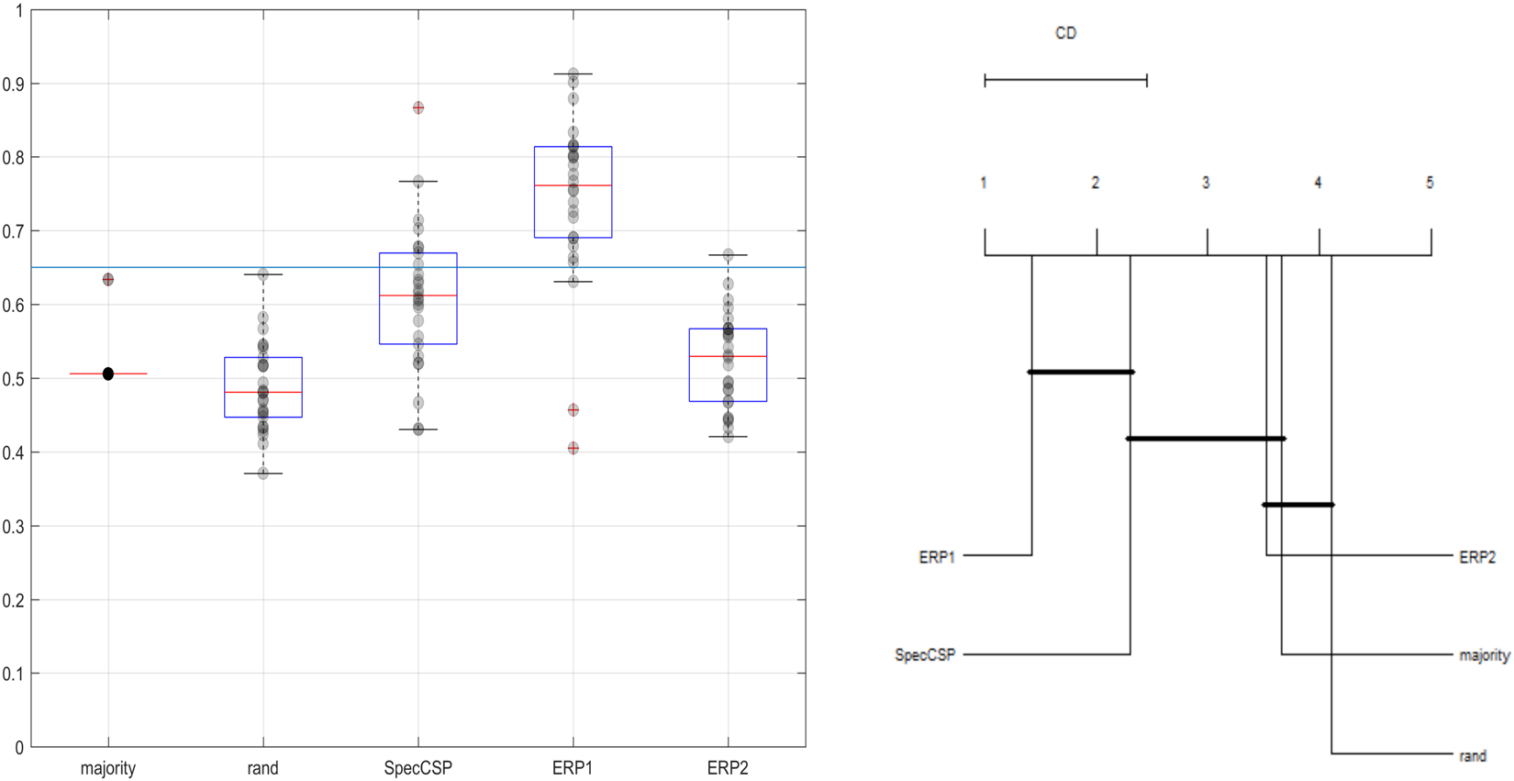
*Left*. Classification of observation tasks (during cue display) of the two visual categories. *Right*. Comparison of all classifiers against each other with the Nemenyi test. Groups of classifiers that are not significantly different are connected.

**Figure 2:**
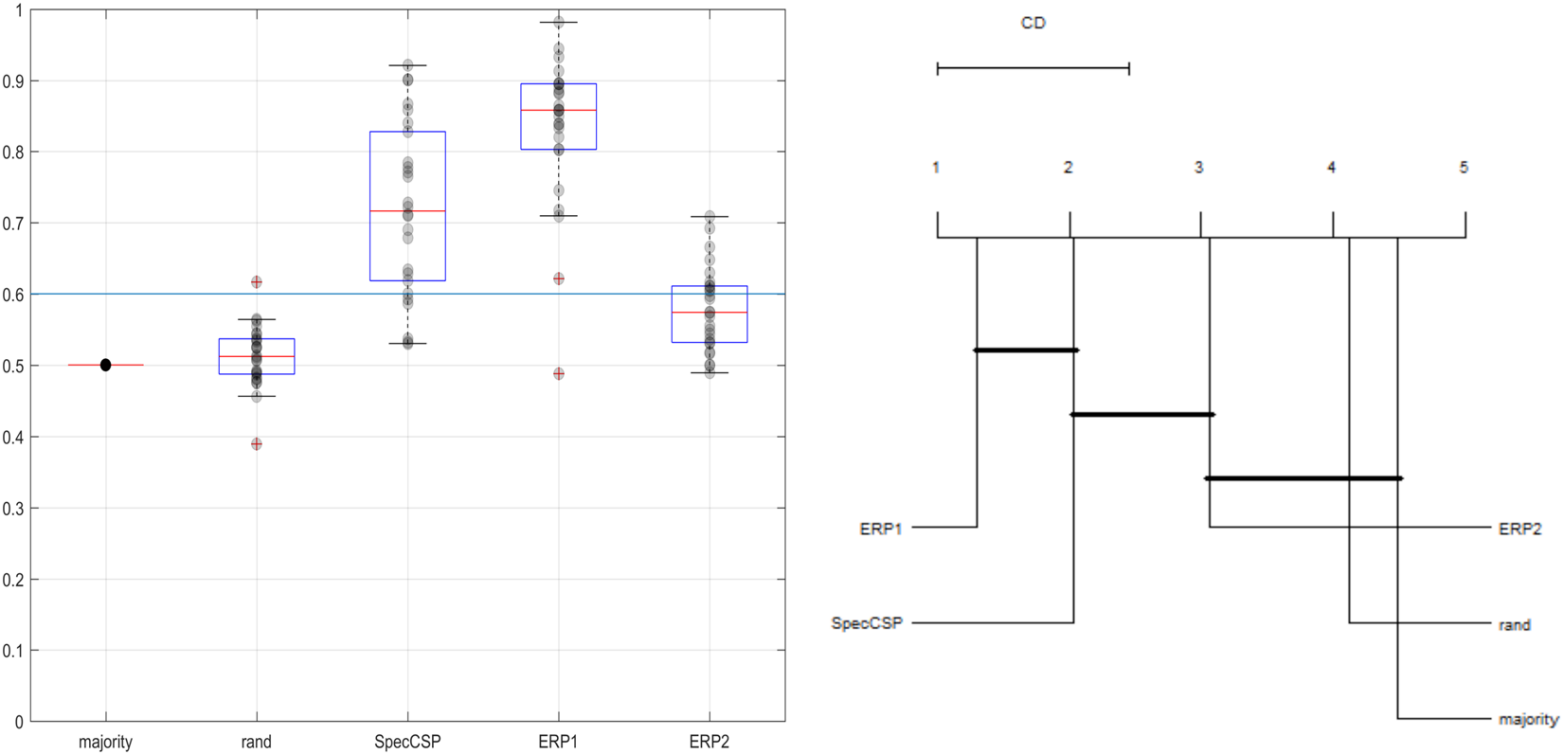
*Left*. Classification of observation task versus rest. *Right*. Comparison of all classifiers against each other with the Nemenyi test. Groups of classifiers that are not significantly different are connected.

The Spectrally Weighted Common Spatial Patterns (SpecCSP) classifier could distinguish between two observation stimuli with around 60% classification accuracy, which however does not exceed the confidence interval upper boundary for the design (65%). In the 2-class classification between the both observation stimuli merged together versus rest, the SpecCSP classifier could distinguish between observation and rest with a 72% classification accuracy (boundary at 60% due to more trials). The results for classifying between the observation stimuli and between observation stimuli and rest are shown in Figs 1 and 2. In the plots on the Figs 1 and 2 and all similar plots in this paper, the red line denotes the median of the cross-validation accuracy across the users for the method, with the upper and lower boundaries of the box denoting the 25% and 75% quantiles, respectively. The strikethrough lines are the upper bounds of the used confidence interval. The red stars denote such accuracies as were considered outliers by the Matlab’s boxplot routine. We compared the five different algorithms using Friedman’s non-parametric rank test with a corresponding post-hoc test^41^. Further, to better understand the possible similarities in the behavior of the tested algorithms, we plotted Critical Difference (CD) images as introduced by Demšar, relying on post-hoc Nemenyi tests^41^ with a threshold alpha = 0.01. In the CD images, two algorithms are not significantly different if they are connected by a bold line. We relied on public R implementations to compute the tests^42^.

The behavior and meaning of the ERP1 and ERP2 classifiers will be discussed later. Please also refer to Table 1, “Observation VS Observation, SpecSCP” column to see the classification accuracy of observation (during cue display) of the two visual categories for each participant presented individually.

**Table 1:** The first column represents the subject number, the second column - classification accuracy of two imagined visual categories, and the third column - classification accuracy of observation (during cue display) of the two visual categories.

Subject number	Imagery VS Imagery, SpecCSP, %	Observation VS Observation, SpecCSP, %
1	0.4833	0.4667
2	0.6333	0.8667
3	0.6333	0.8
4	0.5431	0.5778
5	0.5319	0.5194
6	0.5306	0.4306
7	0.5167	0.6403
8	0.5542	0.6056
9	0.5306	0.5194
10	0.5444	0.5458
11	0.7153	0.7667
12	0.5056	0.5556
13	0.6889	0.6319
14	0.4806	0.6153
15	0.6403	0.5958
16	0.6667	0.7139
17	0.5069	0.6764
18	0.5069	0.7028
19	0.6194	0.6292
20	0.5181	0.6694
21	0.4819	0.5292
22	0.5556	0.6778
23	0.5681	0.6194
24	0.4806	0.6542
25	0.5097	0.6083
26	0.5319	0.4319
Mean value	0.5594	0.6096

### Classification of observation versus imagery task

**Figure 3:**
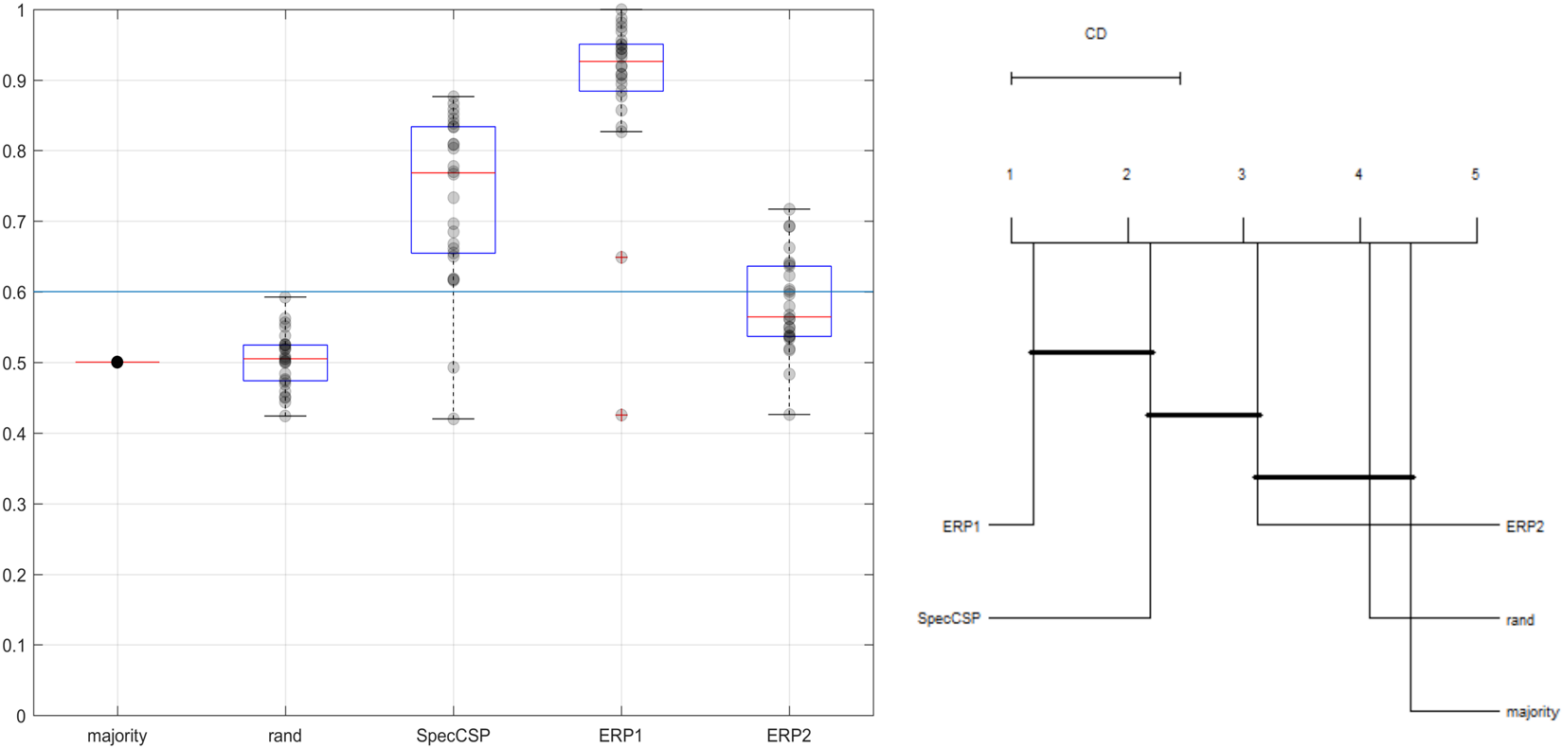
*Left*. Classification of observation-merged task (during cue display) versus imagery-merged task. *Right*. Comparison of all classifiers against each other with the Nemenyi test. Groups of classifiers that are not significantly different are connected.

The SpecCSP classifier could distinguish between observation-merged task versus imagery-merged task with 71% of classification accuracy (confidence boundary at 60%). The results for classifying between the imagery categories and between observation stimuli are shown in Fig. 3. Thus, our null hypotheses H1 and H3 can be rejected.

### Classification of visual imagery tasks

**Figure 4:**
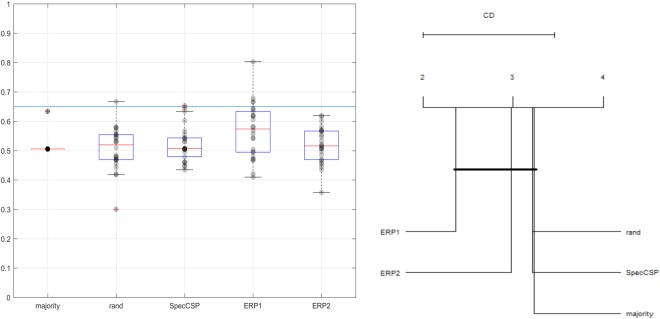
*Left*. Classification of two imagined visual categories. *Right*. Comparison of all classifiers against each other with the Nemenyi test. Groups of classifiers that are not significantly different are connected.

**Figure 5:**
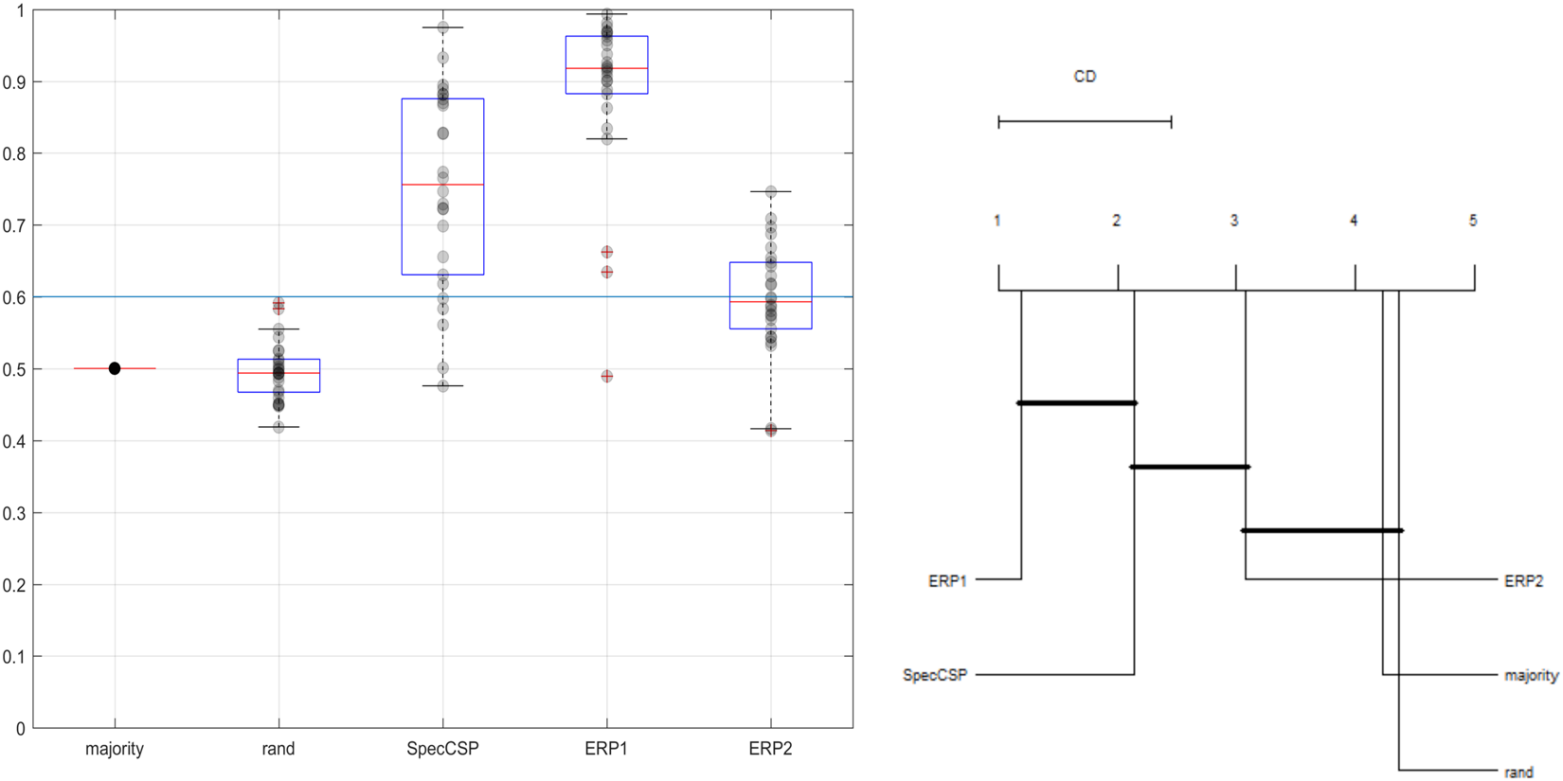
*Left*. Classification of imagery-merged task versus rest. *Right*. Comparison of all classifiers against each other with the Nemenyi test. Groups of classifiers that are not significantly different are connected.

**Figure 6:**
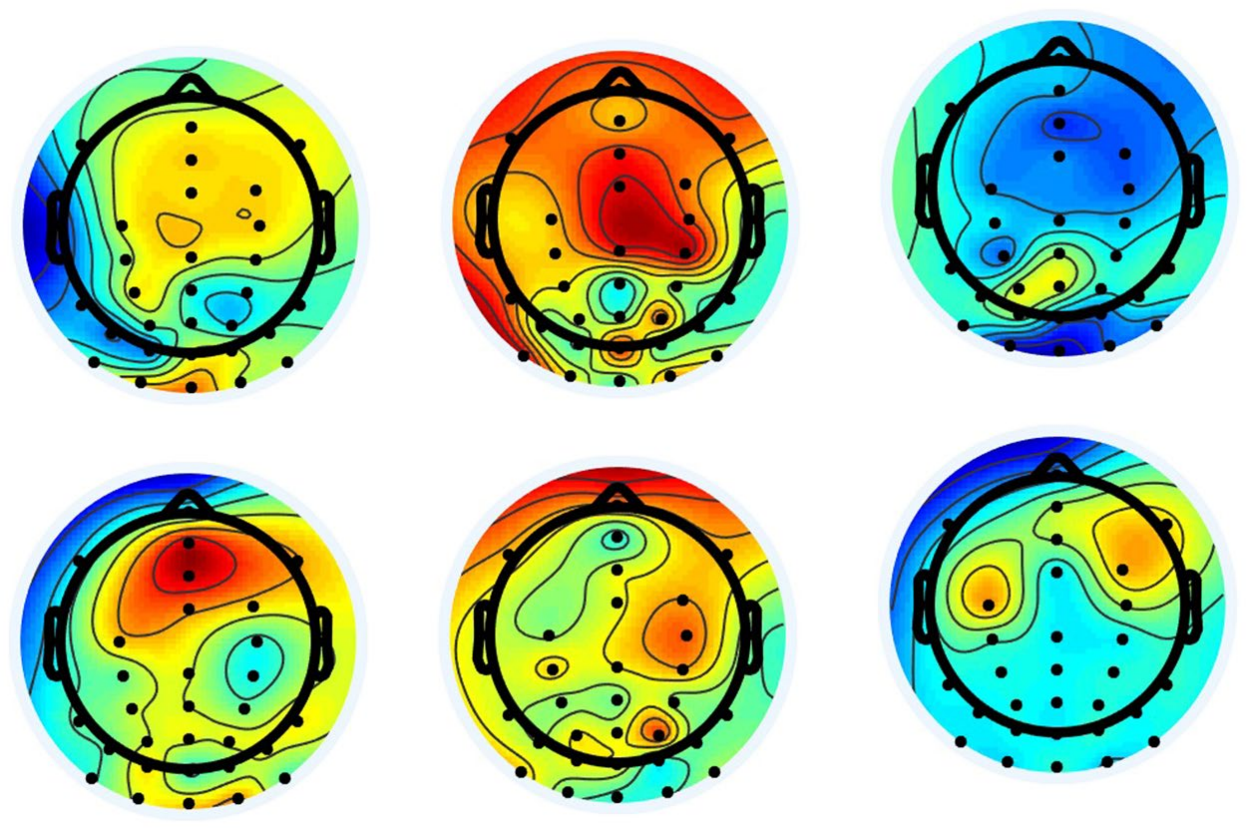
Spectrally Weighted Common Spatial Pattern Maps; a set of SpecCSPs filters of a single participant in the study, who obtained 65% of classification accuracy. The SpecCSPs are computed for the discrimination of visual imagery of the flower from visual imagery of the hammer.

The SpecCSP pipeline could distinguish between two imagery tasks with 52% classification accuracy, which is under the 65% confidence bound. In the 2-class classification between the imagery-merged task versus rest, the SpecCSP pipeline could distinguish between the two with the 77% classification accuracy (with confidence bound at 60%). The results for classifying between the imagery categories and between imagery-merged task and rest are shown in Figs 4 and 5. Please also refer to Table 1, “Imagery VS Imagery, SpecSCP” column to see the classification accuracy of two imagined visual categories for each participant presented individually. Figure 6 illustrates a set of SpecCSPs filters of a single participant in the study, who obtained 65% of classification accuracy. The SpecCSPs are computed for the discrimination of visual imagery of the flower from visual imagery of the hammer.

These results indicate that although it is difficult to tell the two imagery categories apart by the pipelines we are using, as the result is close to random one, the imagery segments are nevertheless distinguishable from the rest condition. Thus, the null hypothesis H2 cannot be rejected, but H4 can be rejected.

### Reliability of the classification results

In addition to comparing the classification pipelines against two trivial random classifiers and the confidence bounds, we implemented a few other controls to investigate if we could find simple, alternative explanations to the obtained accuracies.

The ERP1 and ERP2 pipelines using the Window Means algorithm were run for different time segments, ERP1 for [0, 0.8]s and ERP2 for ^1,3^s, the latter like the other pipelines. The results indicate that switching from condition to condition in the experimental timeline does create event related potentials that can be discriminated from each other to some extent. I.e. if the condition is rest, the rest-onset can be discriminated from cue-display or imagery condition onsets. If the condition is observation, the cue type can be discriminated to some extent as well. The type of imagery could be predicted with much lower accuracy. However, after 1 s, this ERP-type activity has largely ceased and ERP2 working on a later time segment provides much lower accuracies. This suggests that the results obtained from the tested methods during the ^1,3^s segment may not be simply due to stimulus-locked ERP activity resulting from transitions from one condition to another.

The chronological cross-validation in BCILAB mitigates accuracy biases from time-correlated EEG to some extent. To further test that the presented results are not trivially due to some slow temporal change of brain state during our experiment, we additionally split a trial of each type to two segments which were ^1,2^s and ^2,3^s after stimulus onset. Then, we considered these two segments as separate classes to discriminate; for example, first half of cue-display of hammer versus second half of cue-display of hammer. We tested the SpecCSP pipeline with various balanced, 2-class pairings, and obtained per-recording cross-validation accuracies from 40% to 64%. This suggests that tiny accuracy improvements over random can be obtained in our setting even if the underlying categories are not different. The implication is that the possible drift of the brain state that may segregate the first part of each trial from the second part is not well exploitable by the pipelines we test. The contrary would have given evidence that the classifiers are indeed able to use temporal drifts in the EEG.

One possible criticism is that in our experimental design, the sequence of events is always repeated in the same order. In principle it could be possible that a specific trial is discriminable because of some pre-condition residual remains in the EEG until the actual condition. A proper way to control for this would be designing a new experiment where the condition order is perfectly randomized.

### ERD/S patterns in alpha band

Difference between the observation and imagery tasks was reflected in their ERD/S patterns.

**Figure 7:**
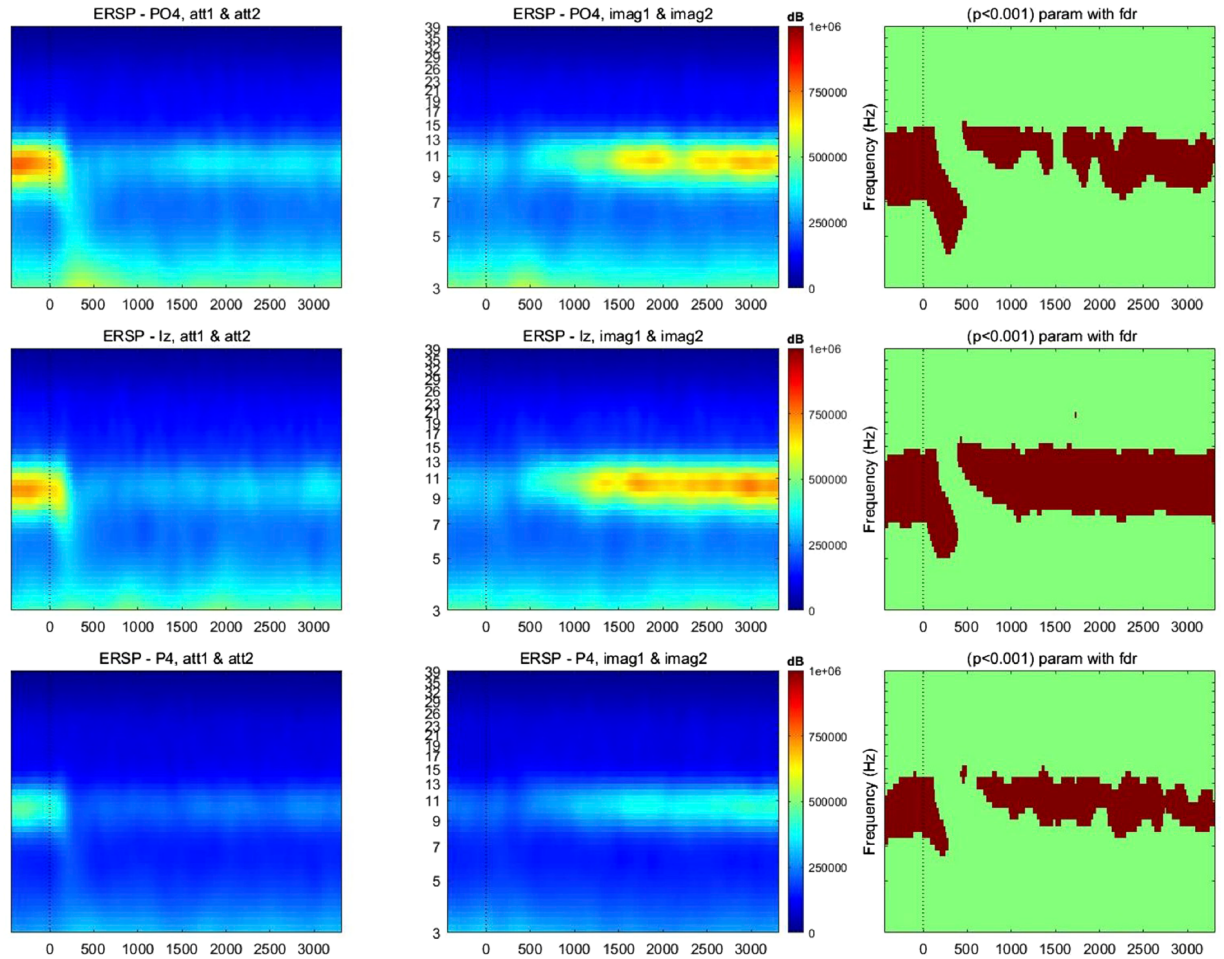
Examples of ERSP visualization of channels PO4, Iz, P4. Alpha activity being significantly higher during the imagery task than during the observation task. In each ERSP image, the abscissa represents the time (−500 to +3000 ms), while the ordinate represents the frequency (from 3 to 40 Hz), the red color indicates power increase with respect to the baseline (dB), while the blue color the power decrease (dB).

Alpha activity is significantly higher during the imagery than during the observation. This significance is strong. ERPs are significant on all the channels on the visual area and though all the time windows, beginning-end of the trial; Event Related Spectral Perturbations (ERSPs) are significant for all channels on the visual area (Fig. 7).

**Figure 8:**
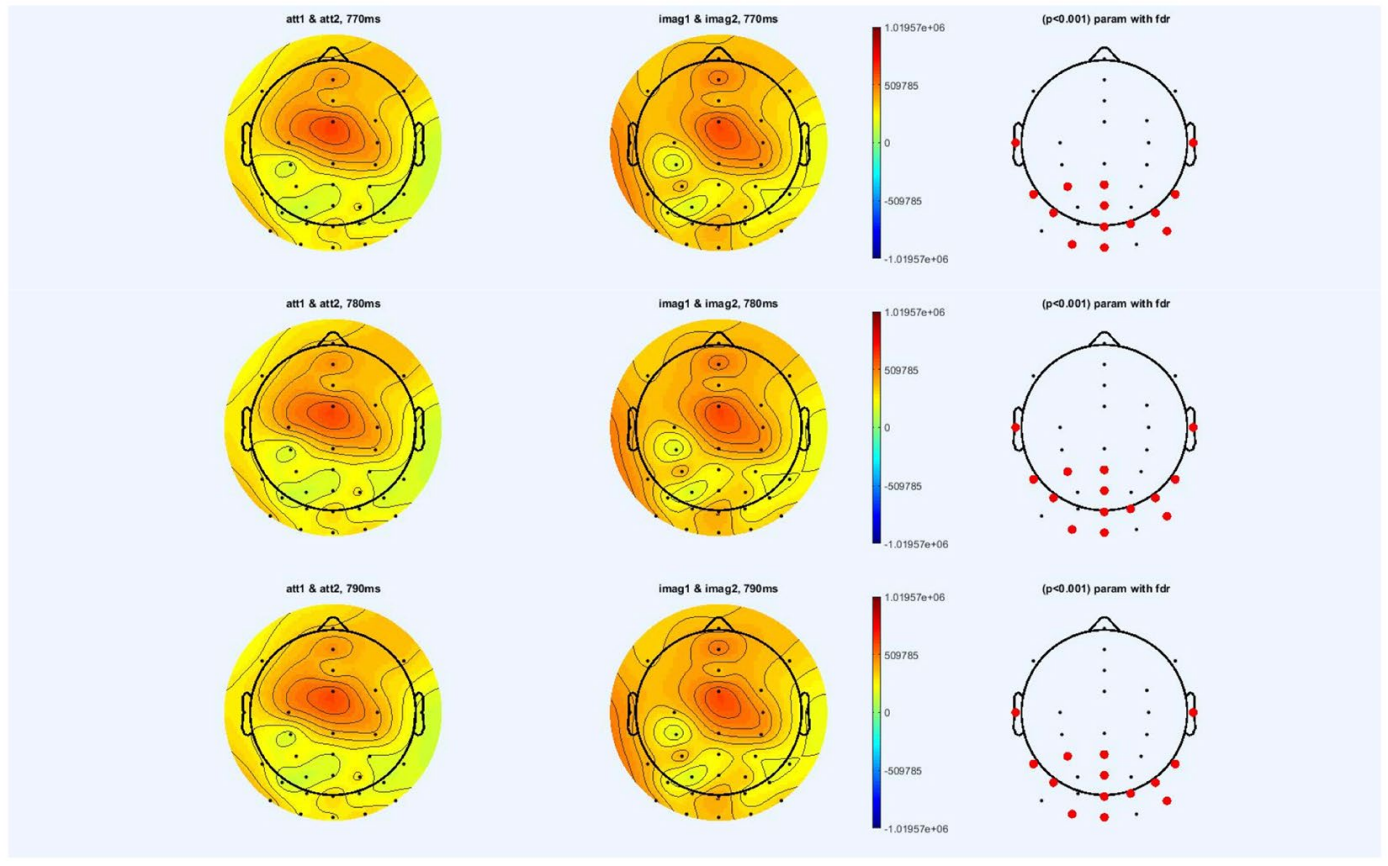
Alpha activity is significantly higher during the imagery task than during the observation task, here from 770 ms till 830 ms. The black dots represent the electrode positions.

Significant main effects for observation versus imagery were observed in all channels on the visual area (Fig. 8). This finding goes in line with the work of^33^ that showed that alpha activity was significantly higher during the imagery process than during the observation one. This finding contradicts the “idling theory” that alpha power desynchronizes when users are engaged when performing cognitively demanding tasks. Klimesch^43^ reported a “synchronization of alpha activity during the retention period in a short term memory task”. Synchronization of alpha activity was also reported by various studies^29,30,36,44,45^ during different cognitive tasks such as mental imagery, memorization tasks, so on.

## Discussion

In the current study we explored the potential of Visual Imagery for BCIs. We studied to what extent can we distinguish two different visual imagery tasks using EEG-based Brain-Computer Interfaces to provide a broader range of reliable control tasks.

Firstly, for the observation-merged task versus imagery-merged task, we obtained the classification accuracy of 73%. This finding is further confirmed by a clear difference of alpha amplitude between imagery and observation stimuli.

Our results confirm the state of the art studies reporting that “increasing alpha desynchronization for higher memory load or task complexity was found for tasks which require constant externally directed observation, while findings for increasing alpha synchronization for higher memory load (or task complexity) have been obtained in tasks which relate to retention and manipulation of internally represented information (i.e., top-down processing)”. Moreover, we could distinguish between two observation stimuli with around 60% classification accuracy. In summary, these findings suggest that observation stimuli could be further investigated for usage in a BCI paradigm. This suggestion is further supported by another finding of our experiment, as we obtained the classification accuracy of 77% between imagery task (for both stimuli) versus resting state, and the classification accuracy of 75% between observation task (for both stimuli) versus resting state. Collectively, these results are promising, as they demonstrate the potential of a BCI system with one “active” state corresponding either to imagery or observation task (as in^39^).

As for the imagery versus imagery task, we obtained the classification accuracy of 52%. The difference to random accuracy is small, and this could be due to several reasons. Firstly, the selected images of hammer and daisy may be difficult to discriminate from the resulting EEG, regardless of technique. There is currently no study using these images that would prove otherwise. The reasons could be due to the nature how the neural assemblies work in the imagery case, due to volume conduction, etc. This limitation may even be user-specific taking into consideration our limited number of participants (10–30% of users cannot obtain any control using mental imagery BCIs^46^). Secondly, the problem might be due to our signal processing pipelines. We did not try to find alternative features to represent the data, but used well-known feature types from previous BCI studies. Another possible reason for our classification results are due to the naivety of our subjects. Almost none of them have performed a mental imagery task with BCIs before, and they only underwent a single recording session, which is not the case for most of studies that present mental imagery tasks, where users are asked to perform multiple sessions of training and recordings^5,47^. It should be also mentioned that we obtained the classification accuracy of around 60% for half of the subjects in our study (see Table 1), and these subjects could be seen as candidates for whom a further improvement may be possible with additional training sessions as suggested in Dyson *et al*.^48^. Although^48^ suggested this approach for motor imagery paradigm, it could be a beneficial strategy for any mental imagery approach. Although we have chosen the images from different semantic categories, several studies^23,24^ showed that visual response is segregated by semantic category. We consider that the obtained results could be explained without invoking a notion of semantic categorization. The obtained result could be related to the selectivity to certain visual attributes that differentiate the two images (a rounded form of the flower, an elongated form of the hammer), as in^22,49^. Moreover the two images we have chosen for our study could be considered as “ecologically” user-friendly, although this introduces one of the limitations: the images were not controlled for the visual attributes such as contrast, color distribution, total amount of lightness, covered volume of visual field, etc.^25^ showed that if two classes of visual stimuli have a low amount of within-class variation, it is possible to get around 60% of correct classification.

## Improving Bcis Using Visual Imagery?

In this study we found that it is possible to discriminate: (i) presence of visual imagery *vs*. observation, (ii) observation task towards different visual stimuli and (iii) rest vs. observation/imagery. We thus conclude that presence of visual imagery and specifically related alpha power changes are useful to broaden the range of reliable BCI strategies. But how could we use these findings to improve BCIs?

Consider BCIs we have nowadays, which are applied to different domains and applications: communication (yes/no; spellers; web browser navigation); environmental control (wheelchair control, robotics); recreation (games); creative expression (music, visual art); attention monitoring; meditation training; computational user experience (mental workload); coma detection and rehabilitation.

The nature of the BCI control rarely corresponds to the semantics of the task; for example, Galan *et al*^50^. propose to use word association task to turn the wheelchair right; and there are more examples of this type, as the most well studied BCI paradigm for active control remains motor imagery. BCIs usually do not highlight the directness of the mapping between the user’s mental activity (e.g., imagining a melody) and the semantics of the task carried by the system (telephone rings). Such lack of logical connection may impact the performance of the task drastically^51^. A broader range of imagery strategies could help developing a broader range of reliable BCI-based control applications. Consider a BCI that detects motor imagery poorly, but reliably detects visual imagery. This BCI might be only 50% accurate if the user tries to communicate via motor imagery but 65% accurate if the user imagines an object he/she wants to manipulate. In that case the BCI could be re-configured to rely on visual imagery. Having more options for BCI control is an opportunity for users for whom classical, state of the art imagery approach (e.g. currently - motor imagery) BCIs are not successful, as it could provide them with a possibility of having another try with another type of imagery. Nowadays, this option is not much studied in the research laboratories. To the best of our knowledge, there are only works that propose to replace one limb-type of motor imagery by another (e.g., arm imagined movement is replaced by foot imagined movement^52,53^, but not one type of active imagery by another one, as the choices are very limited. The question of applications is also rising: if a user is navigating in the space, motor imagery is more preferable than visual one, but if he/she wants to use a coffee machine, than the latter could be of higher interest by imagining a cup of coffee, for example.

## Study Limitations and Future Work

Our study has several limitations that need to be pointed out. As such, we can anticipate several directions of future research efforts to overcome the limitations of the present study:

- Other images should be tested as the stimuli, both close semantically but also in visual properties, such as form and color, in order to investigate the degree of generalizability of the studied paradigm.
- The types of extracted features and used classifiers should be further studied to find which suit the visual modality best.
- Further investigation should be performed to study the use of observation task and/or alpha activity as a potential BCI control paradigm. We plan to conduct another experiment, where the stimuli will be present to the participants during the whole duration of the trial (during observation *and* imagery tasks), so it will be possible to confirm if we are able to disentangle the imagery process from the observation process.
- The current study and the obtained results should be considered as *preliminary*. We encourage the community to pursue the research efforts on investigating in more depth the visual imagery as a control signal for BCIs in general but also to replicate the current experiment to gather more insight and data for further analysis.

More generally, using visual imagery paradigm and visual observation tasks could make BCI-based systems more realistic with relation to particular tasks and more representative when it comes to evaluating the actual performance (imagining/looking at a telephone ringtone to answer a phone versus a hand movement imagery). These types of systems would go in the direction of user-centered design, essential to the design of real-life systems and yet little developed for BCIs. However, to confirm this statement, we would need to run additional online, real-time experiments.

## Methods

### Participants

This study included 26 right-handed participants (7 women), between 18 and 45 years old, without any reported disabilities. All had normal or corrected-to normal vision. Each participant gave informed consent to the study. The experiment was approved and carried out in accordance with the relevant guidelines, regulations and authorizations of Inria Rennes (where actual study took place) and with respect of the Declaration of Helsinki. 20/26 participants had never participated in BCI experiment before. 6 participants tried BCIs for the second time.

### Stimuli

**Figure 9:**
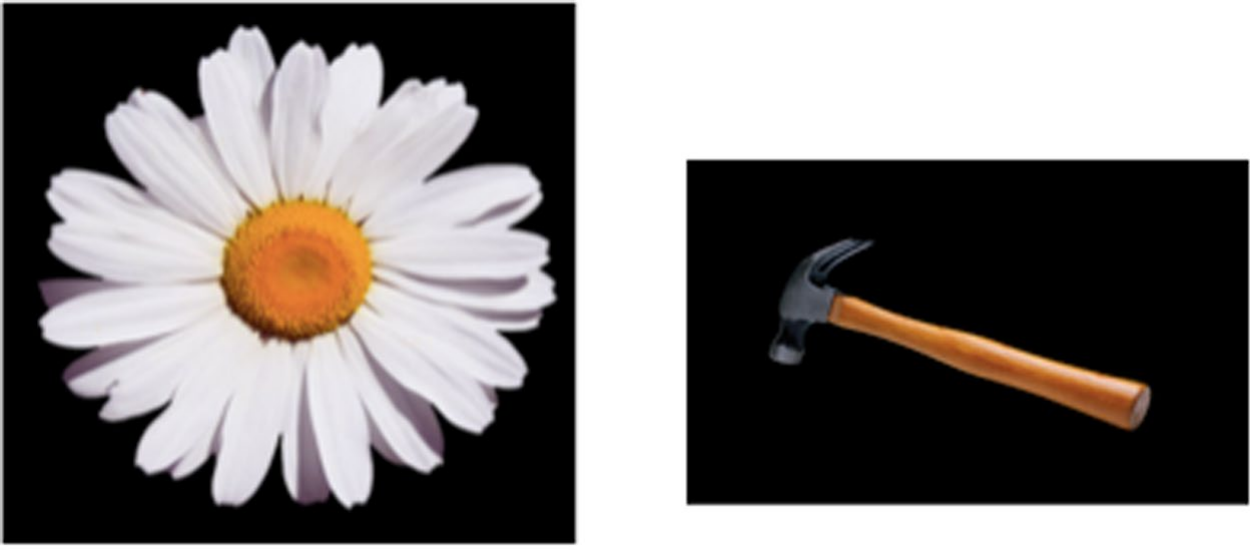
Two visual stimuli used in the current experiment: a flower and a hammer.

Visual stimuli were two images: a hammer and a daisy (Fig. 9). The rationale behind this choice was the following: we took the images of the same size from different semantic categories (tool, nature), which had different color and form and concept of use. We wanted the images to be different, as it might correspondingly increase discriminable differences in the EEG data. Moreover these two images could be considered as “ecologically” user friendly (images come from the environment and not generated artificially), although this introduces one of the first limitations of the present study: the images used in the study are not controlled for the visual attributes such as contrast, color distribution, total amount of lightness, covered volume of visual field.

**Figure 10:**
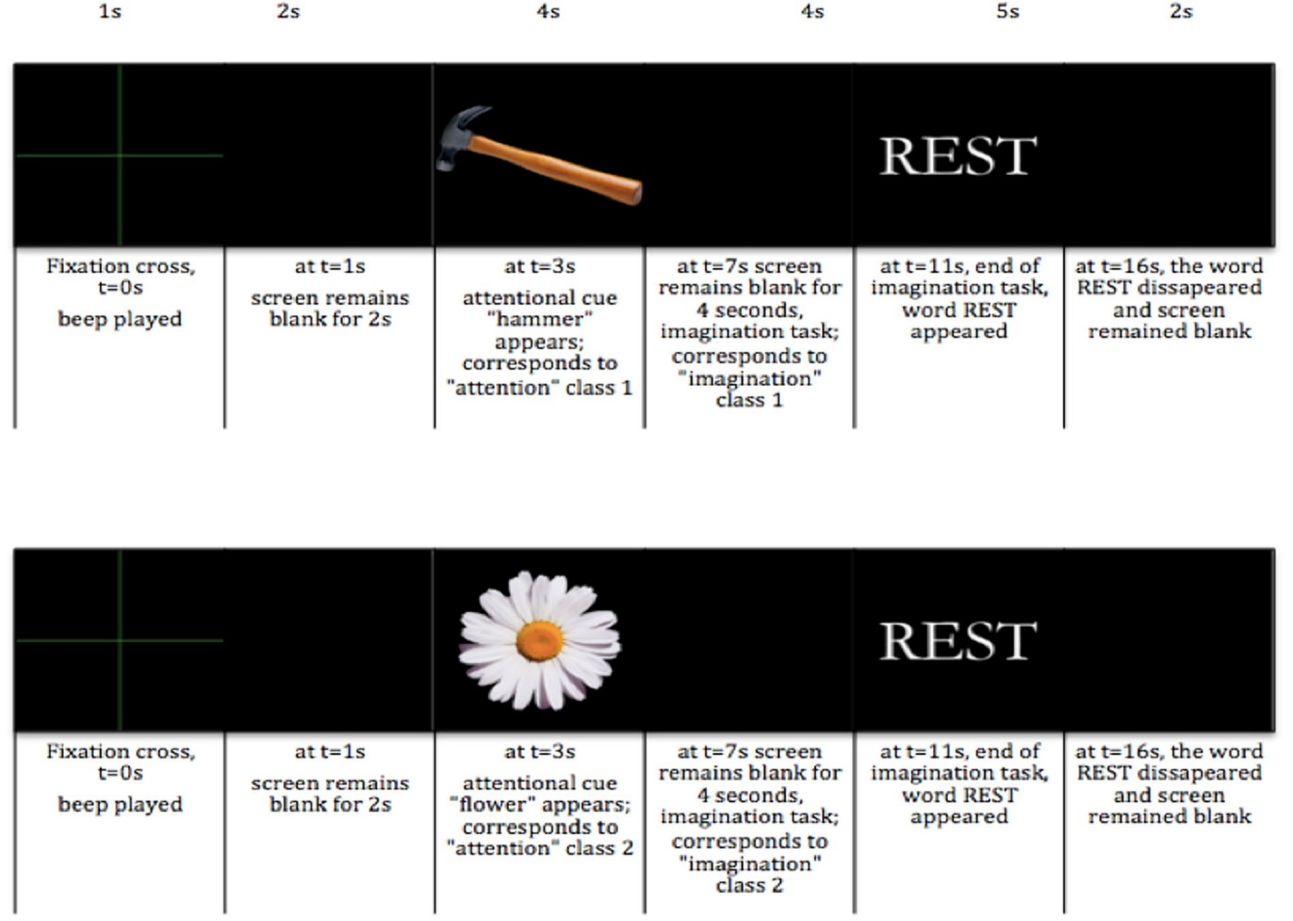
Experimental paradigm with two possible visual stimuli, a hammer and a flower. The duration of the each phase of the trial is indicated above the image, the description of what was happening during each moment of time is indicated below the image.

Our experimental protocol is based on the standard Graz BCI training protocol^48^. Details on the experimental paradigm are summarized in Fig. 10. Users were asked to perform successively a visual observation and a visual imagery task for 4 seconds each while staying motionless. At t = 0 s, a fixation cross was presented on the screen (baseline) for 1 second together with an audible beep. The screen remained blank for 2 seconds after the cross disappeared. At t = 3 s, one of two cues was displayed for 4 seconds. Once the cue disappeared, the screen remained blank for another 4 seconds, during which the users were asked to perform an imagery task for 4 seconds. At t = 11 s, a word REST appeared and remained on the screen for 5 seconds. It indicated the end of the trial. Finally, at t = 16 s, the word REST disappeared and the screen remained blank till the beginning of the new trial. Before the beginning of the very first trial, a baseline of 30 seconds was recorded. After each 10^th^ trial there was a pause with a duration between 120 and 240 seconds.

We make sure that the same observation cue is never presented two times in a row. Please refer to the video of the paper to see the experimental paradigm.

Users were asked to perform the mental task following these instructions:

1. *Visual observation task*: observe carefully the image you see in front of you;
2. *Visual imagery task*: imagine the image that was previously displayed.

Before users performed the task, they completed an overt exercise run: the users observed the two cue images and then they were asked to imagine them. The purpose of this exercise run was to familiarize the participants with the task and to ensure that they understood it and performed it according to the instructions.

During the periods when the word REST appeared on the screen, the users were instructed not to perform any type of imagery/observation tasks.

### Procedure, EEG Recordings and Evaluation of Tasks

We used the electrode configuration of Iz, P7, P8, CP3, CPz, T7, T8, O1, Oz, O2, P3, Pz, P4, CP4, F7, C4, FC4, FC3, C3, FCz, F3, Fz, F8, POz, O9, O10, PO4, PO3, AFz, FPz, Cz, PO8, P8, PO10, PO7, PO9 using 2 g.tec USBAmp amplifiers. Each electrode was referenced to the left and grounded at the right mastoid. The data was filtered (0.5–100 Hz; 50 Hz notch filter), amplified and digitized (512 Hz). The whole experimental set-up (including the instruction, EEG montage, and 1 h of EEG recordings) lasted 1h20. The participants were instructed not to move. The stimuli were presented on a Dell notebook with a 15.4” TFT monitor that was positioned horizontally (flat on a table). The viewing distance was approximately 50 cm from the eyes to the center of the visual display.

This was a within-subject experiment design where the total number of trials was 80 (40 trials per class, and hence 80 in cases where two trial types are merged).

To record the data, we used an open source software OpenViBE^54^.

### Signal processing

We used the freely available EEGLAB and BCILAB packages^55^ for the data analysis and classification, respectively. Note that the data was processed in two ways (*data analysis* and *classification*) and these two processing types do not have the same signal processing pipeline. In *data analysis* we wish the data to be as clean as possible for interpretability. However, as one of the motivations of our study was investigating the visual imagery for EEG-based BCIs, online BCI classification has to make predictions on the trials as they are received. Expert intervention or offline clean-up stages cannot be used. For this reason, we used BCILAB classification pipelines as they were defined in BCILAB, directly on the raw data.

The same amount of data was used for each user in c*lassification*. No trials or channels were removed for *classification* (BCILAB, including SpecCSP patterns). For generating ERSPs/ERPs (in EEGLAB), e.g., for *data analysis*, most users have different amount of trials.

#### Data analysis

We first bandpass filtered the signal to a frequency range of 0.1 Hz–40 Hz using a Hamming windowed FIR band-pass filter from EEGLAB and then removed the eye artifacts using Infomax Independent Component Analysis (ICA)^56^. For each separate recording, we visually identified 0–2 ICA components corresponding to strong, frontally localized activity patterns and removed these components from the signal by setting them to zero, followed by reconstruction of the signal. Note that the present recordings did not seem to have many during-trial eye-artifacts, possibly due to the instructions to the users. Next, we epoched the data to 4.875 second segments starting one second before each stimulus (event) onset. The last 125 ms were removed from each epoch simply to avoid overlapping event markers in the epoched data. Next, we used an iterative algorithm to remove the outlying trials and channels from each recording. The algorithm worked as follows: a high initial threshold for acceptable signal range was set, and then all epochs were marked as outliers that exceeded the specified range. If more than 20% of the epochs per channel were marked as rejected, then the channel itself was rejected. The process was iterated with a little lower threshold each time while disregarding previously rejected channels and epochs until the total number of rejected epochs exceeded 10% of the total epoch count for the dataset. The rejected epochs were dropped from further consideration and the rejected channels were restored by spherical interpolation of nearby electrodes using routines available in EEGLAB. With our data, this procedure rejected around 0–3 electrodes per dataset and around 10% of the trials in each.

For computing time/frequency power plots (ERSP, in EEGLAB), we removed the Event Related Potentials (ERPs) from the epochs by mean subtraction in the time/voltage representation. Note that we have not done any other baseline removal or normalization for the displayed plots (Figs 5 and 6), as the present objective is to examine if differences are present between the two conditions in general.

#### Classification

In addition to statistical analysis of the EEG ERSP features, we also investigated classification of the trials of different types. For this, we applied classifiers from the BCILAB toolbox^55^. Here we used the recorded data directly without discarding any trials or channels. We used only such preprocessing as the BCILAB classifier paradigms do by default (e.g. bandpass filtering). Out of the methods provided by the BCILAB toolbox, we chose the Spectrally Weighted CSP (SpecCSP)^57^ and the Window Means paradigms (ERP2^58^). We selected two-second long segments of each trial to represent the trial, each segment starting 1 second after the event onset to avoid possible transition effects. As the Window Means classifier is usually used to classify ERP patterns, we also used it during a [0, 800]ms segment after stimulus onset (ERP1).

SpecCSP method run with a wide spectral band of 2.5–30 Hz of the signal. This was done to provide the methods with relatively loose spectral priors. The wide band allows the Spectrally Weighted CSP the possibility to select a tighter spectral weighting in that range. For the Window Means (ERP classifiers), we used 0.1–15 Hz band with six consecutive time windows, each 50 ms long and starting at 250 ms after the segment start. We used Logistic Regression as the classification technique for SpecCSP pipeline.

We also created two trivial classification pipelines, one predicting the training set majority class label (‘majority’) and the other outputting random predictions using class weights from the training fold class label distribution (‘rand’). In several plots, we include the results of these control pipelines. The main intent is to visualize to what extent the real classification pipelines deviate from random baseline behavior.

The accuracies we report were obtained by using 10-fold chronological cross-validation from BCILAB, with the margin width set to 5 trials. We used the chronological cross-validation to avoid nearby trials ending up in both train and test folds. We did this in order to reduce possible bias due to time-correlations between neighboring EEG trials.

To double-check if the classification results are significantly different from what could be achieved by chance, we follow Muller-Putz *et al*.^59^. They propose that the reported accuracies should be compared against the upper bound of a confidence interval around the expected accuracy of a random classifier. Accuracy averages inside the confidence interval cannot be rejected from being essentially random. The interval bounds depend on the amount of trials in the experimental design. In the accuracy plots we present, a horizontal line denotes the upper boundary for the confidence interval with a threshold α = 0.01 as obtained from Muller-Putz *et al*.^59^.

## Conclusion

This study investigated the use of Visual Imagery for Brain-Computer Interfaces. We studied to what extent can we distinguish mental processes of observing and imagining two different stimuli in visual perception using EEG-based BCI system. 26 subjects were instructed to alternatively and selectively observe one of two visual stimuli (a flower or a hammer) and mentally imagine one of them following a visual cue. We investigated the EEG correlates of these tasks and analyzed the classification accuracies for visual imagery versus observation of visual stimuli but also versus rest. We found that it is possible to discriminate: (i) visual imagery vs observation task, (ii) observation directed towards different visual stimuli and (iii) rest vs. observation/imagery. We thus conclude that presence of visual imagery and specifically related alpha power changes are useful to broaden the range of reliable BCI control strategies.

## Data Availability

The datasets generated during and/or analyzed during the current study are available from the corresponding author on request.
